# Impact of statin therapy on late target lesion revascularization after everolimus-eluting stent implantation according to pre-interventional vessel remodeling and vessel size of treated lesion

**DOI:** 10.1007/s00380-022-02104-0

**Published:** 2022-06-20

**Authors:** Kohei Asada, Teruki Takeda, Yosuke Higo, Yuichi Sawayama, Noriaki Yagi, Megumi Fukuyama, Masayuki Yamaji, Hiroshi Sakai, Hiroshi Mabuchi, Takashi Yamamoto, Yoshihisa Nakagawa

**Affiliations:** 1 Department of Cardiovascular Medicine, Shiga University of Medical Science, Seta tsukinowa-cho, Otsu, Shiga 520-2192 Japan; 2 Department of Cardiovascular Medicine, Koto Memorial Hospital, Higashiomi, Japan; 3 Department of Cardiovascular Medicine, Kohka Public Hospital, Kohka, Japan

**Keywords:** Coronary disease, Revascularization, Everolimus-eluting stent, Restenosis, Statin therapy

## Abstract

Clinical evidence demonstrating the impact of statins for preventing late target lesion revascularization (TLR) after newer-generation drug-eluting stent implantation and differences in the effect of statins on late TLR according to pre-interventional vessel remodeling and vessel size is limited. We retrospectively evaluated 1193 de novo lesions in 720 patients who underwent everolimus-eluting stent implantation using intravascular ultrasound from January 2010 to December 2012. The primary endpoint was late TLR. Lesions were divided into the statin group (*n* = 825) and non-statin group (*n* = 368). The incidence of late TLR was significantly lower in the statin than non-statin group (1.7% vs. 5.2%, respectively; *p* = 0.001), and within the statin group, it was significantly lower in the follow-up low-density lipoprotein cholesterol (LDL-C) < 100 than ≥ 100 mg/dL level subgroup (1.0% vs. 3.6%, respectively; *p* = 0.006). Furthermore, in positive remodeling lesions and non-small vessel size lesions, the incidence of late TLR was significantly lower in the statin than non-statin group (1.6% vs. 8.5% and 1.3% vs. 5.3%, respectively;* p* = 0.001 and *p* = 0.004). Lowering the LDL-C level using statins was more effective for preventing late TLR after everolimus-eluting stent implantation. Evaluating pre-interventional vessel remodeling patterns and vessel size might be helpful to stratify lesions at high risk of late TLR.

**Supplementary Information:**

The online version contains supplementary material available at 10.1007/s00380-022-02104-0.

## Introduction

Despite improvements in percutaneous coronary intervention (PCI) techniques and advancements in newer-generation drug-eluting stent (DES) technology and adjunctive medical therapy, late target lesion revascularization (TLR) beyond 1 year after DES implantation still exists, and the prevalence of late TLR is not negligible. It is now understood that some cases of late TLR arise from the pathogenic concept of neoatherosclerosis. Recent studies of histopathology and intracoronary imaging have shown that neoatherosclerotic changes within the stented segment are characterized by accumulation of lipid-laden foamy macrophages, and such changes have been identified as a primary cause of delayed failure such as late TLR [1–4]. Although the precise mechanisms of neoatherosclerosis have not been elucidated, the low-density lipoprotein cholesterol (LDL-C) level and underlying unstable plaques are considered to be associated with neoatherosclerosis after stent implantation [2, 5]. A high LDL-C level is associated with atherosclerosis progression and cardiovascular adverse events. Accordingly, current guidelines now recommend more aggressive pursuit of a low LDL-C level using high-intensity statins for secondary prevention [6–8]. However, whether the use and intensity of statin therapy are associated with the prevention of late TLR using newer-generation DES technology remains unknown. Therefore, we analyzed the effect of statin therapy and the influence of the achieved LDL-C level on late TLR after everolimus-eluting stent (EES) implantation using 4-year follow-up data.

We also tested the hypothesis that the characteristics of underlying plaques are associated with an increase in late TLR. A pathological study showed that coronary artery plaques that undergo positive remodeling have a significantly larger lipid core and a higher macrophage count than those that undergo vessel shrinkage [9]. Furthermore, a larger vessel size is associated with a higher lipid content of coronary plaques [10]. Therefore, we investigated the impact of pre-interventional vessel remodeling and the vessel size of treated lesions on late TLR.

## Materials and methods

### Study population

**Fig. 1:**
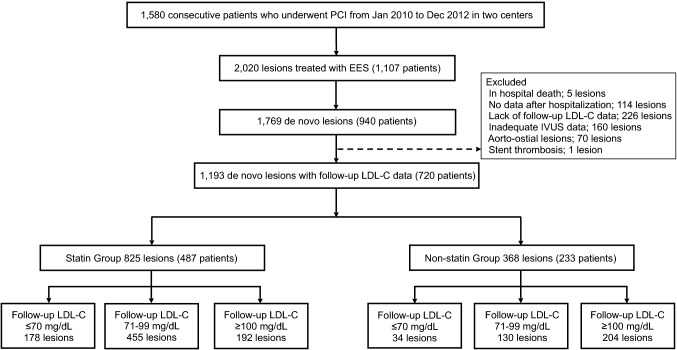
Flow chart of the study. *PCI* percutaneous coronary intervention, *EES* everolimus-eluting stent, *IVUS* intravascular ultrasound, *LDL-C* low-density lipoprotein cholesterol

This retrospective cohort study was performed to evaluate the incidence of TLR after EES implantation for de novo coronary artery lesions from January 2010 to December 2012 in two centers (Shiga University of Medical Science Hospital and Koto Memorial Hospital). Patients who underwent implantation of at least one EES were included in the study, regardless of whether they had received stents other than an EES. The study flow chart is shown in Fig. 1. Of the initial 1580 consecutive patients who underwent PCI, EES implantation was performed in 1769 de novo lesions of 940 patients during 3 years of enrollment. Excluding patients with in-hospital death, loss to follow-up, aorto-ostial lesions, and inadequate intravascular ultrasound (IVUS) data, and stent thrombosis, the remaining 720 patients (1193 lesions) constituted the final study population. The patients were divided into 2 groups according to the use of statins at discharge: 487 patients with 825 lesions who underwent treatment by statin therapy (statin group) and 233 patients with 368 lesions who did not undergo treatment by statin therapy (non-statin group) and evaluated by intention-to-treat analysis. The statin group consisted of 685 lesions (83%) treated by strong statin [atorvastatin, rosuvastatin, and pitavastatin] and 140 lesions (17%) treated by standard statin [pravastatin, simvastatin, and fluvastatin]. A lesion was defined as the area covered by single or multiple overlapping stents. Multiple lesions within the same patient were considered to be independent observations for lesion-specific analyses.

To evaluate the association between the follow-up LDL-C level and late TLR, both the statin group and non-statin group were subdivided into three groups according to the follow-up LDL-C level and in accordance with practice guidelines: the ≤ 70, 71–99, and ≥ 100 mg/dL subgroups. Furthermore, we evaluated the association between the follow-up LDL-C level and late TLR according to pre-interventional vessel remodeling and the vessel size based on IVUS data. Therefore, the lesions were divided into two groups according to pre-interventional vessel remodeling and the vessel size.

The study was conducted in accordance with the Declaration of Helsinki and the Good Clinical Practice standard, and it was approved by the ethics committee at each institution. We obtained consent from all individual participants in the study using an opt-out procedure.

### Data collection and follow-up

Demographic, clinical, angiographic, procedural, and outcome data were obtained from the hospital records or databases at each institution. Follow-up information was obtained from hospital charts or by contacting the patients or their referring physicians.

The follow-up coronary angiography plan was left to the discretion of each referring physician; routine follow-up angiography was not performed. The angiographic follow-up or coronary computed tomography angiography follow-up rate was 84.6%, and the median follow-up duration was 387 days (interquartile range, 356–516 days).

Clinical follow-up data were retrospectively evaluated at 4 years, and follow-up intervals were calculated from the day of the index PCI procedure. The median follow-up duration was 1460 days (interquartile range, 1149–1460 days). Follow-up LDL-C data after the index procedure were retrospectively obtained from the electronic database. When several follow-up LDL-C data were available in a given patient, we used the measurement closest to 1 year after PCI. Follow-up serum lipid levels were optionally measured, and the median interval from the index procedure to the measurement of the LDL-C level was 352 days (interquartile range, 306–383 days).

### Stent implantation procedure and postprocedural management

Lesions were treated with standard interventional techniques, including mandated balloon dilatation before stent implantation, if required. The duration of dual antiplatelet therapy was left to the discretion of each referring physician. For statin therapy, we did not set indications for the use of statins, and the type of statin was left to the discretion of each referring physician.

### IVUS imaging and analysis

IVUS imaging procedures were performed before the coronary intervention and immediately after the guide wire crossed the lesion or pre-balloon dilatation if the IVUS catheter could not pass the lesion. All IVUS images were obtained after the intracoronary administration of nitrates using a 20-MHz, 2.9-Fr IVUS imaging catheter (Eagle Eye; Volcano Corp., San Diego, CA, USA). The cross-sectional areas (CSA) of the external elastic membrane (EEM), lumen, and stent were measured at the minimum lumen site and reference segments. The proximal reference was defined as the site with the largest lumen proximal to a stenosis and the distal reference site was defined as the site with the largest lumen distal to a stenosis. The remodeling index (RI) was defined as lesion EEM CSA/average of the proximal and distal reference segment EEM CSA, and the patterns of pre-intervention vessel remodeling were classified into two categories: positive and negative remodeling, defined as a RI > 1.00 and RI ≤ 1.00, respectively [11]. Vessel size was classified into two categories by the proximal reference diameter: small and non-small vessel size lesions, defined as a < 3.0- and ≥ 3.0-mm proximal reference lumen diameter based on IVUS information, respectively, with reference to the previous definition [12].

### Clinical outcomes and definitions

The primary outcome in this study was late TLR assessed during the 4-year follow-up interval after EES implantation. TLR was defined as repeat PCI or coronary artery bypass grafting due to restenosis of the target lesion in association with recurrent angina and/or evidence of myocardial ischemia. Early TLR was defined as TLR performed within 1 year after stent implantation, and late TLR was defined as TLR performed > 1 year after stent implantation. Retreatment for stent thrombosis was not considered part of TLR. Death, cardiovascular death, myocardial infarction, stroke, and target vessel revascularization were also assessed as endpoints.

### Statistical analysis

Categorical variables are reported as number and percentage and were compared using the chi-square test or Fisher’s exact test. Continuous variables with a normal distribution are expressed as mean ± standard deviation. Variables with a non-normal distribution are expressed as median and interquartile range. Continuous variables with a normal distribution were compared using Student’s *t* test. The Wilcoxon rank-sum test was used for comparisons of non-normally distributed continuous variables. The cumulative incidence was estimated with the Kaplan–Meier method, and differences were assessed with the log-rank test. To evaluate the impact of statin therapy and the follow-up LDL-C level on late TLR, we used the landmark analysis at 1 year. We used a Cox proportional hazard model to identify the independent risk factors for early and late TLR separately. The variables included in the Cox multivariable regression analysis were age, sex, diabetes mellitus, hemodialysis, statin therapy, stent length of > 28 mm, lesion bending, ostial right coronary artery stenting, ostial left circumflex stenting, and a minimum stent area of < 5.0 mm^2^ with reference to previous reports [13, 14]. We also performed the Cox multivariable regression analysis using same variables in the positive remodeling group and no small vessel size lesions to show importance of statin for late TLR. Furthermore, the adjusted risk for late TLR according to the follow-up LDL-C level in overall and statin group was estimated by the Cox proportional hazard model by incorporating the follow-up LDL-C level < 100 mg/dL together with the above variables excluded of statin. A *p* value of < 0.05 was considered statistically significant. All data were analyzed using JMP version 16.0 software (SAS Institute, Inc., Cary, NC, USA).

## Results

### Patient, lesion, and procedural characteristics

Among the study population of 720 patients with 1193 lesions, 39.4% patients were of advanced age (> 75 years), 40.2% had diabetes mellitus, and 1.8% were undergoing hemodialysis. The patients presented with either stable angina (1128 lesions) or acute coronary syndrome (65 lesions) (Table 1).

**Table 1:** Characteristics of patients with lesions treated by everolimus-eluting stents

Variables	Overall lesions (*n* = 1193)	Lesions in statin group (*n* = 825)	Lesions in non-statin group (*n* = 368)	*p* value
Age, years	71.1 ± 9.49	70.0 ± 9.69	73.5 ± 9.03	< 0.0001
Age of ≥ 75 years	470 (39.4)	289 (35.0)	181 (49.2)	< 0.0001
Male sex	879 (73.7)	592 (71.8)	288 (78.0)	0.02
Coexisting conditions				
Hypertension	900 (75.4)	611 (74.1)	289 (78.5)	0.10
Diabetes mellitus	479 (40.2)	339 (41.1)	140 (38.0)	0.32
Hemodialysis	21 (1.8)	8 (1.0)	13 (3.5)	0.002
Estimated glomerular filtration rate of < 30 mL/min/1.73 m^**2**^	42 (3.5)	20 (2.4)	22 (6.0)	0.002
Current smoker	336 (28.2)	241 (29.2)	95 (25.8)	0.24
Previous myocardial infarction	297 (24.9)	234 (28.4)	63 (17.1)	< 0.0001
Previous percutaneous coronary intervention	700 (58.7)	491 (59.5)	209 (56.8)	0.38
Previous coronary artery bypass grafting	19 (1.6)	12 (1.5)	7 (1.9)	0.57
Previous stroke	122 (10.2)	80 (9.7)	42 (11.4)	0.37
Clinical characteristics				
Acute coronary syndrome	65 (5.5)	55 (6.7)	10 (2.7)	0.006
Left ventricular ejection fraction of < 40%	110 (9.2)	65 (7.9)	45 (12.2)	0.02
Multivessel disease	779 (65.3)	549 (66.6)	230 (62.5)	0.18
Baseline medications				
Dual antiplatelet therapy	1116 (93.6)	777 (94.2)	339 (92.2)	0.18
Aspirin	1180 (98.9)	819 (99.3)	361 (98.1)	0.07
Thienopyridine	1150 (96.4)	801 (97.1)	349 (94.8)	0.054
Warfarin	68 (5.7)	42 (5.1)	26 (7.1)	0.17
Beta blocker	285 (23.9)	206 (25.0)	79 (21.5)	0.19
ACE-I or ARB	739 (61.9)	533 (64.6)	206 (56.0)	0.005
Mineralocorticoid receptor antagonist	94 (7.9)	67 (8.1)	27 (7.3)	0.64
Eicosapentaenoic acid	81 (6.8)	54 (6.6)	27 (7.3)	0.62
Baseline lipid levels				
Total cholesterol, mg/dL	185.1 ± 38.2	185.8 ± 41.0	183.4 ± 30.9	0.32
High-density lipoprotein cholesterol, mg/dL	47.4 ± 12.8	48.0 ± 12.6	46.1 ± 13.4	0.02
Triglycerides, mg/dL	147.8 ± 82.6	148.9 ± 79.7	145.3 ± 88.7	0.49
Low-density lipoprotein cholesterol, mg/dL	108.9 ± 30.1	109.5 ± 32.4	107.6 ± 24.2	0.32
Achieved lipid levels				
Total cholesterol, mg/dL	166.3 ± 30.4	161.7 ± 30.1	178.4 ± 31.2	< 0.0001
High-density lipoprotein cholesterol, mg/dL	49.6 ± 13.3	49.9 ± 13.4	48.8 ± 13.0	0.23
Triglycerides, mg/dL	144.6 ± 86.2	147.4 ± 91.3	137.4 ± 71.3	0.10
Low-density lipoprotein cholesterol, mg/dL	92.9 ± 24.2	87.8 ± 23.3	104.1 ± 26.2	< 0.0001
Target vessel location				
Left main coronary artery	12 (1.0)	10 (1.2)	2 (0.5)	0.36
Left anterior descending coronary artery	463 (38.8)	305 (37.0)	158 (42.9)	0.051
Left circumflex coronary artery	309 (25.9)	208 (25.2)	101 (27.5)	0.42
Right coronary artery	408 (34.2)	301 (36.5)	107 (29.1)	0.01
Lesion and procedure characteristics				
ACC/AHA Type B2/C	1012 (84.8)	706 (85.6)	306 (83.2)	0.28
Moderate or heavy calcification	718 (60.2)	497 (60.3)	221 (60.1)	0.93
Chronic total occlusion	48 (4.0)	36 (4.4)	12 (3.3)	0.37
Lesion bending	210 (17.6)	146 (17.7)	64 (17.4)	0.90
Eccentric	388 (32.5)	275 (33.3)	113 (30.7)	0.37
True bifurcation	341 (28.6)	243 (29.5)	98 (26.6)	0.32
Thrombus	81 (6.8)	70 (8.5)	11 (3.0)	0.001
Total number of stents	1.28 ± 0.53	1.29 ± 0.54	1.27 ± 0.51	0.59
Total stent length, mm	27.9 ± 15.9	28.4 ± 16.1	26.6 ± 15.4	0.07
Total stent length of > 28 mm	326 (27.3)	234 (28.4)	92 (25.0)	0.23
Lesion length of > 20 mm	586 (49.1)	426 (51.6)	160 (43.5)	0.01
Stent size, mm	2.92 ± 0.38	2.92 ± 0.38	2.91 ± 0.38	0.51
Minimum stent size of < 3.0 mm	584 (49.0)	396 (48.0)	188 (51.1)	0.32
Direct stenting	599 (50.2)	399 (48.4)	200 (54.4)	0.06
Postdilatation	1183 (99.2)	818 (99.2)	365 (99.2)	0.95
Ostial LCX stenting	21 (1.8)	14 (1.7)	7 (1.9)	0.80
Ostial RCA stenting	8 (0.7)	5 (0.6)	3 (0.8)	0.71
Bifurcation 2-stent approach	45 (4.0)	29 (3.7)	16 (4.5)	0.55

Data are presented as mean ± standard deviation or *n* (%)

*ACC* American College of Cardiology, *ACE-I* angiotensin-converting enzyme inhibitor, *AHA* American Heart Association, *ARB* angiotensin II receptor blocker, *LCX* left circumflex artery, RCA right coronary artery

### Clinical outcomes: statin versus non-statin

**Fig. 2:**
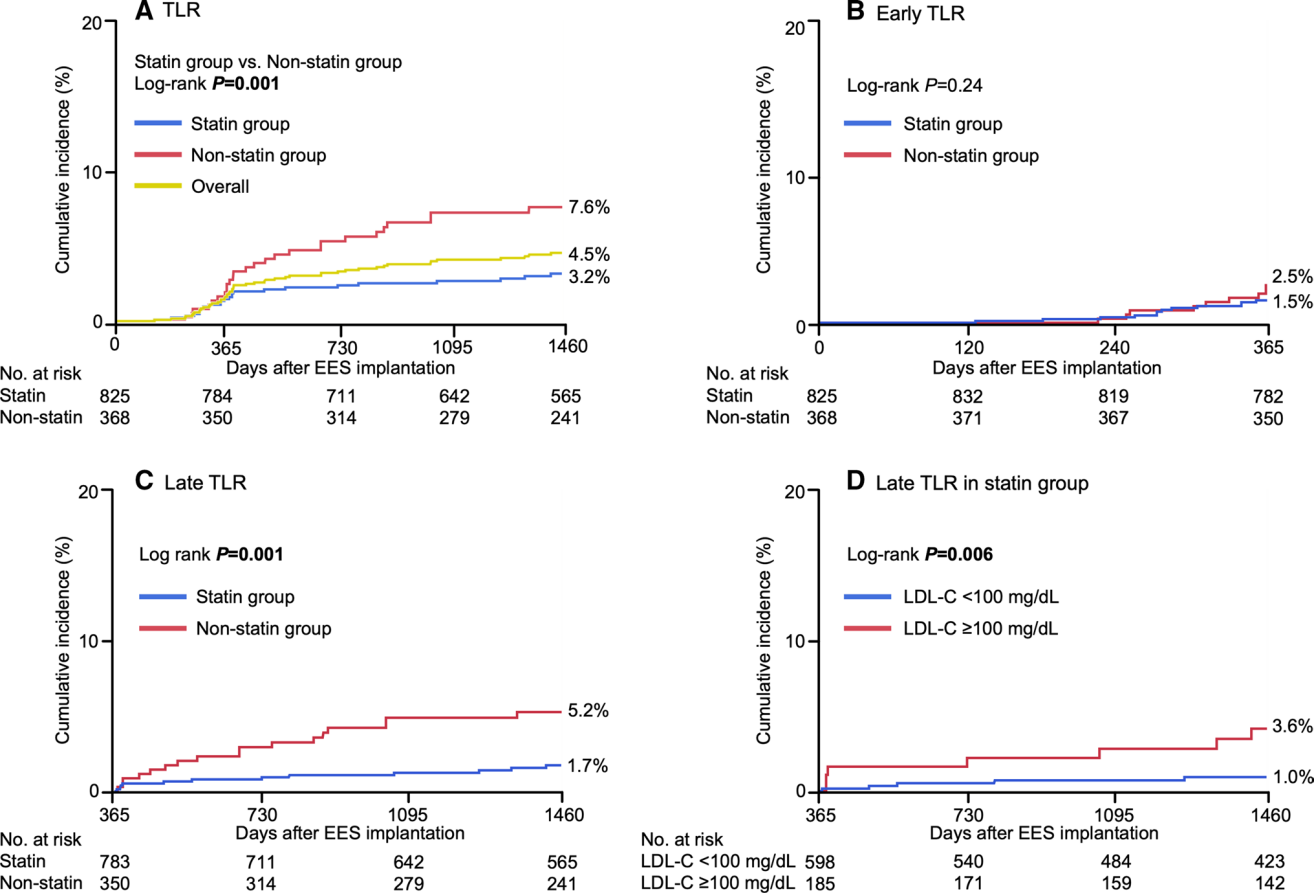
Incidence of **A** TLR, **B** early TLR, and **C** late TLR in the statin versus non-statin groups. **D** Incidence of late TLR in the statin group in the follow-up LDL-C ≥ 100 versus < 100 mg/dL subgroups (LDL-C ≤ 70 + 71–99 mg/dL subgroups). *EES* everolimus-eluting stent, *LDL-C* low-density lipoprotein cholesterol, *TLR* target lesion revascularization

During the 4-year follow-up period, the cumulative incidence of TLR in the overall lesions was 4.5% (Fig. 2), that of early TLR was 1.8%, and that of late TLR was 2.7%. Late TLR continuously occurred up to 4 years. The cumulative 4-year incidence of TLR was significantly lower in the statin group than in the non-statin group (3.2% and 7.6%, respectively; *p* = 0.001). The incidence of early TLR was not significantly different between the statin and non-statin groups (1.5% and 2.5%, respectively; *p* = 0.24), whereas the incidence of late TLR was significantly lower in the statin group than in the non-statin group (1.7% and 5.2%, respectively; *p* = 0.001) (Fig. 2). After adjusting for confounding factors by a Cox regression model, the risk for early TLR was comparable between the two groups [hazard ratio (HR), 0.60; 95% confidence interval (CI), 0.25–1.42; *p* = 0.24] (Table 2). However, the risk for late TLR was significantly lower in the statin group than in the non-statin group (HR, 0.25; 95% CI, 0.11–0.57; *p* = 0.001) (Table 2). Furthermore, statin use was a protective factor for late TLR even in the positive remodeling group and no small vessel size lesions, respectively (HR, 0.26; 95% CI, 0.09–0.73; *p* = 0.01 and HR, 0.20; 95% CI, 0.06–0.67; *p* = 0.009) (Supplementary Table 1).

**Table 2:** Univariate and multivariate analyses of risk factors for early TLR and late TLR

Clinical Factors	Early TLR						Late TLR					
	Univariate			Multivariate			Univariate			Multivariate		
	HR	95% CI	*p* value	HR	95% CI	*p* value	HR	95% CI	*p* value	HR	95% CI	*p* value
Age of > 75 years	0.62	0.24–1.60	0.33	0.53	0.18–1.56	0.25	1.63	0.79–3.38	0.19	1.09	0.49–2.45	0.83
Sex (male = 1)	0.90	0.35–2.33	0.83	0.69	0.23–2.00	0.48	0.67	0.31–1.43	0.30	0.82	0.35–1.93	0.65
Hypertension	1.05	0.38–2.86	0.93				0.87	0.38–1.96	0.73			
Diabetes mellitus	2.95	1.19–7.30	0.02	2.33	0.90–6.00	0.08	1.85	0.89–3.84	0.10	2.00	0.93–4.27	0.07
Hemodialysis	9.22	2.72–31.3	0.0004	4.49	1.12–18.0	0.03			0.99			0.99
Multivessel coronary disease	0.86	0.36–2.09	0.75				1.24	0.57–2.73	0.59			
Acute coronary syndrome			0.98				0.71	0.10–5.23	0.74			
Type B2/C	3.69	0.50–27.5	0.20				1.67	0.51–5.52	0.40			
Moderate or heavy calcification	1.08	0.45–2.59	0.87				1.24	0.57–2.69	0.58			
Chronic total occlusion	1.27	0.17–9.45	0.82						0.99			
Eccentric	1.05	0.42–2.59	0.92				1.31	0.62–2.79	0.47			
Lesion bending	0.78	0.23–2.65	0.69	0.75	0.22–2.61	0.65	2.93	1.38–6.21	0.005	2.56	1.16–5.62	0.02
True bifurcation	1.26	0.51–3.12	0.62				1.17	0.53–2.56	0.70			
Ostial LCX stenting	6.38	1.49–27.4	0.01	4.96	1.06–23.1	0.04	4.62	1.10–19.4	0.04	4.25	0.94–19.2	0.06
Ostial RCA stenting	8.35	1.12–62.2	0.04	3.70	0.47–29.4	0.22	7.99	1.09–58.7	0.04	4.17	0.50–34.5	0.19
Bifurcation 2-stent approach	2.11	0.28–16.1	0.47				1.22	0.17–9.00	0.85			
Minimum stent size of < 3.0 mm	1.39	0.59–3.30	0.46				0.74	0.35–1.56	0.43			
Total stent length of > 28 mm	4.42	1.83–10.7	0.001	3.71	1.49–9.20	0.005	1.55	0.72–3.34	0.26	1.52	0.67–3.46	0.32
Minimum stent area of < 5.0 mm^**2**^	2.36	0.95–5.85	0.06	1.97	0.78–4.99	0.15	0.90	0.42–1.89	0.77	0.82	0.38–1.77	0.82
Statin	0.60	0.25–1.42	0.24	0.71	0.28–1.80	0.47	0.31	0.15–0.66	0.002	0.25	0.11–0.57	0.001

*TLR* target lesion revascularization, *HR* hazard ratio, *CI* confidence interval, *LCX* left circumflex artery, *RCA* right coronary artery

There were no significant differences in the incidences of all-cause death, cardiac death, myocardial infarction, or stroke between the two groups (Table 3).

**Table 3:** Event rates at 4 years in statin versus non-statin group

Variables	Statin	Non-statin	*p* value
	(*n* = 487)	(*n* = 233)	
All-cause death	21 (4.9)	13 (6.3)	0.52
Cardiovascular death	5 (1.1)	4 (1.9)	0.46
Myocardial infarction	5 (1.1)	1 (0.5)	0.40
Stroke	8 (1.8)	1 (0.5)	0.16
Target lesion revascularization	18 (4.1)	17 (7.9)	0.04
Target vessel revascularization	67 (15.0)	50 (23.3)	0.02

Data are presented as number (%) of events of patients

### Late TLR according to follow-up LDL-C level

No significant differences were found in the baseline lipid profile between the statin and non-statin groups, but the follow-up LDL-C level was significantly lower in the statin group than in the non-statin group (87.8 ± 23.3 and 104.1 ± 26.2 mg/dL, respectively; *p* < 0.001). In the statin group, the risk for late TLR was significantly lower in the follow-up LDL-C level < 100 mg/dL subgroup (≤ 70 + 71–99 mg/dL subgroups) than in the follow-up LDL-C level ≥ 100 mg/dL subgroup (1.0% and 3.6%, respectively; *p* = 0.006) (Fig. 2). In the non-statin group, however, the incidence of late TLR was not significantly different between the follow-up LDL-C level ≥ 100 and < 100 mg/dL subgroups (5.4% and 10.4%, respectively; *p* = 0.08). After adjusting for confounding factors included follow-up LDL-C level < 100 mg/dL by a Cox regression model, the follow-up LDL-C level < 100 mg/dL was associated with a significantly decreased incidence of late TLR in the statin group, whereas the follow-up LDL-C level < 100 mg/dL was not significantly associated in overall (Supplementary Table 2).

**Fig. 3:**
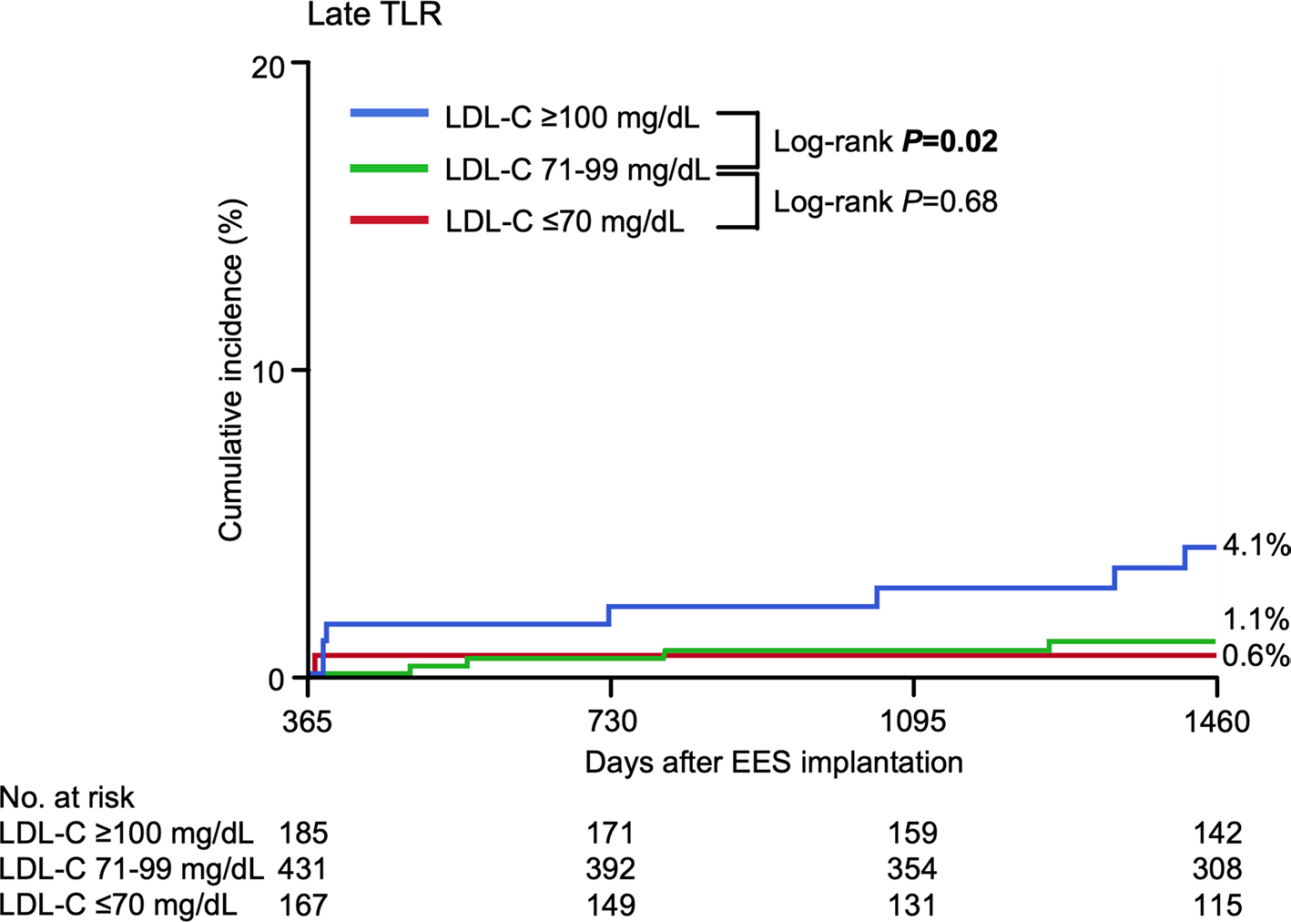
Incidence of late TLR according to follow-up LDL-C levels in statin group. Follow-up LDL-C ≤ 70 mg/dL versus 71–99 mg/dL versus ≥ 100 mg/dL. *LDL-C* low-density lipoprotein cholesterol, *TLR* target lesion revascularization

In the comparison among the three subgroups of follow-up LDL-C levels, the incidence of late TLR was comparable between the follow-up LDL-C level ≤ 70 and 71–99 mg/dL subgroups. However, the incidence of late TLR was significantly higher in the follow-up LDL-C level ≥ 100 than 71–99 mg/dL subgroup (Fig. 3).

### Risk factors for early and late TLR

The results of the multivariable analysis of early TLR and late TLR are shown in Table 2. The significant risk factors for early TLR were hemodialysis (HR, 4.49; 95% CI, 1.12–18.0; *p* = 0.03), a total stent length of > 28 mm (HR, 3.71; 95% CI, 1.49–9.20; *p* = 0.005), and ostial left circumflex coronary artery stenting (HR, 4.96; 95% CI, 1.06–23.1; *p* = 0.04); however, statin use was not significant (HR, 0.71; 95% CI, 0.28–1.80; *p* = 0.47). The risk factors for late TLR were lesion bending (HR, 2.56; 95% CI, 1.16–5.62; *p* = 0.02) and statin use (HR, 0.25; 95% CI, 0.11–0.57; *p* = 0.001).

### Late TLR according to pre-interventional vessel remodeling and vessel size

The IVUS data are summarized in Table 4. No significant differences in the pre-interventional IVUS findings were found between the statin and non-statin groups.

**Table 4:** IVUS measurements

Variables	Overall (*n* = 1193)	Statin (*n* = 825)	Non-statin (*n* = 368)	*p* value
IVUS findings				
Proximal reference, mm	3.1 ± 0.6	3.2 ± 0.6	3.1 ± 0.6	0.21
Lumen area, mm^2^	8.0 ± 3.1	8.1 ± 3.2	7.9 ± 3.0	0.34
EEM area, mm^2^	15.1 ± 5.2	15.2 ± 5.3	15.1 ± 5.1	0.81
Distal reference, mm	2.8 ± 0.6	2.8 ± 0.6	2.8 ± 0.6	0.65
Lumen area, mm^2^	6.2 ± 2.5	6.3 ± 2.5	6.2 ± 2.4	0.82
EEM area, mm^2^	11.5 ± 5.3	11.4 ± 5.3	11.5 ± 5.5	0.77
Culprit lesion				
Lumen area, mm^2^	3.0 ± 1.1	2.9 ± 1.1	3.0 ± 1.0	0.27
EEM area, mm^2^	12.6 ± 5.1	12.6 ± 5.0	12.7 ± 5.5	0.85
Remodeling index	0.96 ± 0.2	0.96 ± 0.2	0.96 ± 0.2	0.82
Plaque area, %	74.4 ± 8.8	74.6 ± 8.8	74.0 ± 8.8	0.29
Minimum stent area, mm^2^	5.6 ± 2.2	5.6 ± 2.2	5.6 ± 2.2	0.65
Minimum stent area of < 5.0 mm^2^	536 (46.1)	368 (45.7)	168 (47.1)	0.66

Data are presented as mean ± standard deviation or *n* (%)

*EEM* external elastic membrane, *IVUS* intravascular ultrasound

**Fig. 4:**
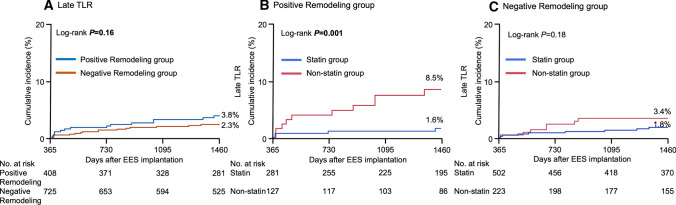
Incidence of late TLR in overall study population: **A** positive remodeling group versus negative remodeling group. Incidence of late TLR in **B** positive remodeling group and in **C** negative remodeling group: statin versus non-statin groups. EES, everolimus-eluting stent; TLR, target lesion revascularization

The positive remodeling group contained 439 (37%) lesions, and the negative remodeling group contained 754 (63%) lesions. In the positive remodeling group, the incidence of late TLR was significantly lower in the statin group than in the non-statin group. In negative remodeling group, however, the incidence of late TLR was not significantly different between the statin group and non-statin group (Fig. 4).

**Fig. 5:**
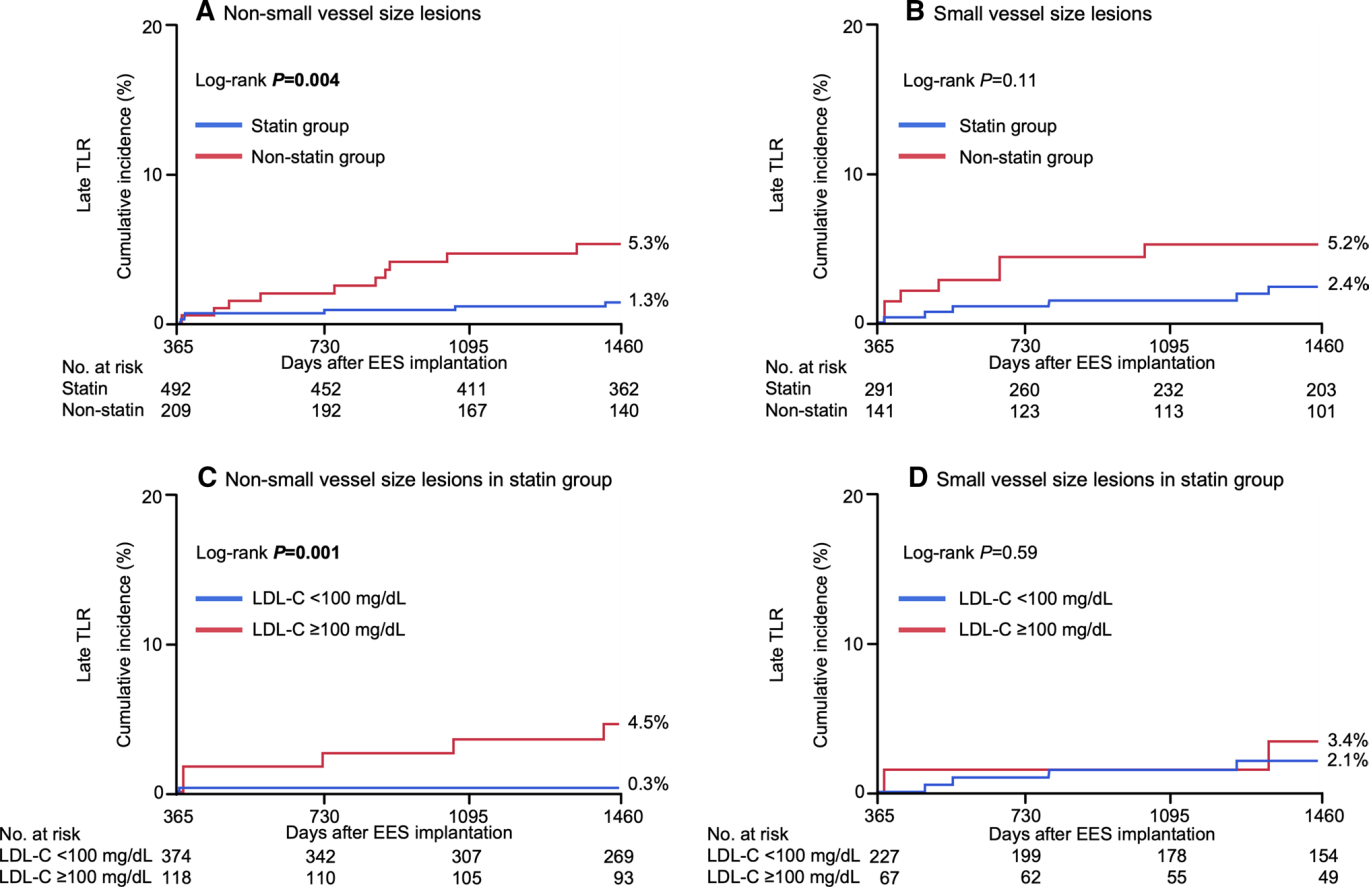
Incidence of late target lesion revascularization in **A** non-small vessel size lesions and **B** in small vessel size lesions: statin versus non-statin groups. In statin group, incidence of late target lesion revascularization according to follow-up low-density lipoprotein cholesterol (LDL-C) level in **C** non-small vessel size lesions and in **D** small vessel size lesions: follow-up LDL-C ≥ 100 versus < 100 mg/dL. *EES* everolimus-eluting stent, *LDL-C* low-density lipoprotein cholesterol

Of all lesions, 731 (61%) were non-small vessel size lesions and 462 (39%) were small vessel size lesions. Among the non-small vessel size lesions, the incidence of late TLR was significantly lower in the statin group than in the non-statin group. Among the small vessel size lesions, however, the incidence of late TLR was not significantly different between the statin and non-statin groups. Within the statin group, the incidence of late TLR in non-small vessel size lesions was significantly lower in the follow-up LDL-C < 100 than ≥ 100 mg/dL subgroup, whereas the incidence of late TLR in small vessel size lesions was not significantly different between the follow-up LDL-C < 100 and ≥ 100 mg/dL subgroups (Fig. 5).

## Discussion

The four main findings of this study can be summarized as follows. First, late TLR after EES implantation occurred without attenuation of the annual incidence up to 4 years, and statin therapy was associated with a significantly decreased incidence of late TLR. Second, a follow-up LDL-C level of ≥ 100 mg/dL was associated with a higher risk for late TLR than was a follow-up LDL-C level of < 100 mg/dL. Third, more severe positive remodeling in the culprit lesion was associated with a greater effect of statin therapy on late TLR. Finally, a larger vessel size in the culprit lesion was associated with a greater effect of statin therapy on late TLR.

### Statins and late TLR

In the present study, late TLR continuously occurred without attenuation up to 4 years, and the incidence rate was similar to those reported in other previous registries evaluating second-generation DES implantation [14, 15]. In the present analysis, statin therapy was associated with a significantly lower rate of late TLR; however, this association was not observed for early TLR, as similarly shown in the Coronary Revascularization Demonstrating Outcome study in Kyoto (CREDO-Kyoto) registry obtained from the data of first-generation DES implantation [16]. These consistent findings suggest that different mechanisms exist for early and late TLR after DES implantation regardless of the generation of the DES. Considering the different effects of statin therapy in the early and late phases, our study indicates that procedural factors might be mainly associated with the progression of neointimal proliferation in the early phase, whereas biological factors, such as persistent inflammation and atherosclerotic change, might be mainly associated with the progression of neointimal proliferation in the late phase. In-stent neoatherosclerosis has been recognized as a primary mechanism of DES failure, particularly late after implantation [17, 18]. Otsuka et al. [19] reported that the observed frequency of neoatherosclerosis did not differ significantly between first- and second-generation DES implantation in a human autopsy study. In-stent neoatherosclerosis remains an unsolved issue even in the era of newer-generation DES therapy. Whether and how clinicians can prevent neoatherosclerosis of a DES remains uncertain. However, given the histological similarities between native atherosclerosis and in-stent neoatherosclerosis, statins might have favorable effects on neoatherosclerosis after DES implantation.

It has been reported that LDL-C reduction therapy using statins can provide better clinical outcomes in ischemic heart disease patients [20, 21], and it is considered essential in secondary prevention of cardiovascular disease. In the present analysis, a lower LDL-C level at follow-up was associated with a lower risk for late TLR in the statin group. A previous optical coherence tomography study revealed a significant relationship between neoatherosclerosis and a higher follow-up LDL-C level in DES-treated lesions [5, 22]. Therefore, intensive LDL-C control therapy might play a significant role in maintaining a favorable neointima pattern and preventing in-stent neoatherosclerosis in the late phase. However, in the non-statin group, the differences in the follow-up LDL-C level were not associated with significant differences in the risk of late TLR. These findings suggest that not only intensive LDL-C lowering, but also administration of statins itself is very important.

### Pre-interventional vessel remodeling, vessel size, and late TLR

Arterial remodeling is defined as geometric alteration of the arterial wall in response to the progression of atherosclerosis. Positive remodeling has been detected more frequently in unstable lesions, and unstable plaques generally have higher lipid content. A previous IVUS study showed a higher lipid content in coronary plaques with positive remodeling and a larger vessel size [10]. Atherosclerotic lesions predominantly occur in large vessels first, and more distal lesions occur with aging. Proximal lesions are usually more evolving, especially with higher rates of unstable plaques in the proximal segments of coronary arteries.

According to a pathological study by Nakazawa et al. [2], the presence of underlying unstable plaques is an independent predictor of neoatherosclerosis. However, evidence connecting neoatherosclerosis with positive remodeling and vessel size is lacking. In the present analysis, statin therapy was more effective for late TLR in positive remodeling lesions. Moreover, statin therapy was more effective in non-small than small vessel size lesions, and intensive LDL-C lowering using statins was more effective for late TLR in non-small vessel size lesions. Lesions with positive remodeling or a large vessel size with a higher lipid content might be more biologically active, and the underlying susceptibility to progressive atherosclerosis may induce neoatherosclerotic change within the neointima as a natural consequence of atherosclerosis. Prediction of neoatherosclerosis by evaluating pre-interventional vessel remodeling and vessel size using imaging modalities might help to stratify lesions susceptible to late stent failure. Results from our study may have important clinical implications for the development and implementation of lipid modification strategies according to pre-interventional vessel remodeling and vessel size to prevent late TLR among patients undergoing PCI.

### Limitations

Our study had several limitations. First, the present study was a retrospective study using data from a non-randomized, observational registry and selection bias in using statins might have affected the results. Second, there was a considerable difference in patients’ and lesions’ background characteristics among groups. Third, we used a less contemporary cohort of patients for our study, which may have affected both the LDL-C target level and rates of statin use compared with a more modern cohort. Fourth, because information about medical therapy was obtained only at hospital discharge, adherence to medications and crossover of medications were not evaluated in this study. Fifth, lesions without IVUS imaging and lesions that could not be evaluated by IVUS imaging because of calcification or ostium lesions were excluded from this analysis. These exclusions might have affected the results. Furthermore, exclusion of patients with no data after hospitalization may have resulted in selection bias.

## Conclusions

In this retrospective study, the lowering LDL-C level using statins was more effective for preventing late TLR after EES implantation. Evaluating pre-interventional vessel remodeling patterns and vessel size using imaging might be helpful to stratify lesions at high risk of late TLR.

## Supplementary Information

Below is the link to the electronic supplementary material.

Supplementary file1 (DOCX 21 KB)

Supplementary file2 (DOCX 21 KB)
