# Supplementary material for: Impact of statin therapy on late target lesion revascularization after everolimus-eluting stent implantation according to pre-interventional vessel remodeling and vessel size of treated lesion
Source: Heart Vessels. 2022 Jun 20;37(11):1817–28. doi: 10.1007/s00380-022-02104-0 (PMC9515046; doi:10.1007/s00380-022-02104-0)
Supplement: Supplementary file 1 — Supplementary file1 (DOCX 21 KB) [file 380_2022_2104_MOESM1_ESM.docx]

**Supplementary Table 1. Univariate and multivariate analyses of risk factors for late TLR in positive remodeling group and no small vessel size lesions**

| Clinical Factors | Late TLR; no small vessel size lesions | | | | | | Late TLR; positive remodeling group | | | | | |
| --- | --- | --- | --- | --- | --- | --- | --- | --- | --- | --- | --- | --- |
|  | Univariate | | | Multivariate | | | Univariate | | | Multivariate | | |
|  | HR | 95% CI | *p* value | HR | 95% CI | *p* value | HR | 95% CI | *p* value | HR | 95% CI | *p* value |
| Age of > 75 years | 1.81 | 0.68–4.83 | 0.24 | 1.44 | 0.52–3.99 | 0.49 | 1.73 | 0.61–4.95 | 0.30 | 1.17 | 0.36–3.80 | 0.79 |
| Sex (male = 1) | 0.69 | 0.24–1.97 | 0.48 | 0.76 | 0.25–2.29 | 0.63 | 0.48 | 0.16–1.42 | 0.18 | 0.63 | 0.18–2.15 | 0.46 |
| Hypertension | 2.36 | 0.54–10.4 | 0.26 |  |  |  | 1.34 | 0.37–4.79 | 0.66 |  |  |  |
| Diabetes mellitus | 0.67 | 0.23–1.94 | 0.46 | 0.74 | 0.26–2.14 | 0.74 | 0.94 | 0.33–2.72 | 0.92 | 1.03 | 0.35–3.05 | 0.96 |
| Hemodialysis |  |  | 0.99 |  |  | 0.99 |  |  | 0.99 |  |  | 0.99 |
| Multivessel coronary disease | 1.85 | 0.60–5.74 | 0.29 |  |  |  | 1.38 | 0.38–4.95 | 0.62 |  |  |  |
| Acute coronary syndrome | 1.17 | 0.15–8.85 | 0.88 |  |  |  | 0.83 | 0.11–6.35 | 0.86 |  |  |  |
| Type B2/C | 1.09 | 0.25–4.82 | 0.90 |  |  |  |  |  | 0.99 |  |  |  |
| Moderate or heavy calcification | 1.92 | 0.62–5.97 | 0.23 |  |  |  | 3.81 | 0.85–17.0 | 0.08 |  |  |  |
| Chronic total occlusion |  |  | 0.99 |  |  |  |  |  | 0.99 |  |  |  |
| Eccentric | 1.55 | 0.58–4.15 | 0.39 |  |  |  | 1.23 | 0.43–3.50 | 0.70 |  |  |  |
| Lesion bending | 3.20 | 1.19–8.59 | 0.02 | 3.09 | 1.11–8.61 | 0.03 | 4.25 | 1.49–12.1 | 0.007 | 3.89 | 1.33–11.4 | 0.01 |
| True bifurcation | 1.83 | 0.68–4.91 | 0.23 |  |  |  | 1.03 | 0.34–3.06 | 0.96 |  |  |  |
| Ostial LCX stenting | 8.97 | 1.18–67.9 | 0.03 | 7.29 | 0.88–60.5 | 0.07 | 7.29 | 1.63–32.6 | 0.01 | 5.58 | 1.10–28.3 | 0.04 |
| Ostial RCA stenting |  |  | 0.99 |  |  | 0.99 |  |  | 0.99 |  |  | 0.99 |
| Bifurcation 2-stent approach |  |  | 0.99 |  |  |  | 1.58 | 0.21–12.1 | 0.66 |  |  |  |
| Minimum stent size of <3.0 mm | 0.87 | 0.28–2.70 | 0.81 |  |  |  | 1.02 | 0.36–2.91 | 0.97 |  |  |  |
| Total stent length of >28 mm | 1.19 | 0.41–3.41 | 0.75 | 1.20 | 0.38–3.77 | 0.75 | 2.08 | 0.73–5.93 | 0.17 | 1.86 | 0.58–6.02 | 0.30 |
| Minimum stent area of <5.0 mm^2^ | 0.97 | 0.31–3.00 | 0.96 | 0.94 | 0.28–3.10 | 0.94 | 1.50 | 0.52–4.31 | 0.46 | 1.25 | 0.40–3.97 | 0.70 |
| Statin | 0.25 | 0.09–0.69 | 0.008 | 0.26 | 0.09–0.73 | 0.01 | 0.18 | 0.06–0.58 | 0.004 | 0.20 | 0.06–0.67 | 0.009 |

TLR, target lesion revascularization; HR, hazard ratio; CI, confidence interval; LCX, left circumflex artery; RCA, right coronary artery.
