# Supplementary material for: Impact of statin therapy on late target lesion revascularization after everolimus-eluting stent implantation according to pre-interventional vessel remodeling and vessel size of treated lesion
Source: Heart Vessels. 2022 Jun 20;37(11):1817–28. doi: 10.1007/s00380-022-02104-0 (PMC9515046; doi:10.1007/s00380-022-02104-0)
Supplement: Supplementary file 2 — Supplementary file2 (DOCX 21 KB) [file 380_2022_2104_MOESM2_ESM.docx]

**Supplementary Table 2. Univariate and multivariate analyses of risk factors for late TLR in overall and in statin group**

| Clinical Factors | Late TLR; overall | | | | | | Late TLR; statin group | | | | | |
| --- | --- | --- | --- | --- | --- | --- | --- | --- | --- | --- | --- | --- |
|  | Univariate | | | Multivariate | | | Univariate | | | Multivariate | | |
|  | HR | 95% CI | *p* value | HR | 95% CI | *p* value | HR | 95% CI | *p* value | HR | 95% CI | *p* value |
| Age of > 75 years | 1.63 | 0.79–3.38 | 0.19 | 1.40 | 0.64–3.06 | 0.40 | 0.71 | 0.19–2.63 | 0.61 | 0.39 | 0.08–1.90 | 0.24 |
| Sex (male = 1) | 0.67 | 0.31–1.43 | 0.30 | 0.86 | 0.37–2.01 | 0.73 | 0.38 | 0.12–1.19 | 0.10 | 0.30 | 0.09–1.06 | 0.06 |
| Hypertension | 0.87 | 0.38–1.96 | 0.73 |  |  |  | 0.73 | 0.22–2.43 | 0.61 |  |  |  |
| Diabetes mellitus | 1.85 | 0.89–3.84 | 0.10 | 1.99 | 0.93–4.26 | 0.08 | 1.42 | 0.46–4.40 | 0.54 | 1.89 | 0.56–6.38 | 0.30 |
| Hemodialysis |  |  | 0.99 |  |  | 0.99 |  |  | 0.99 |  |  | 0.99 |
| Multivessel coronary disease | 1.24 | 0.57–2.73 | 0.59 |  |  |  | 0.73 | 0.23–2.29 | 0.59 |  |  |  |
| Acute coronary syndrome | 0.71 | 0.10–5.23 | 0.74 |  |  |  | 1.46 | 0.19–11.3 | 0.72 |  |  |  |
| Type B2/C | 1.67 | 0.51–5.52 | 0.40 |  |  |  | 0.90 | 0.20–4.10 | 0.89 |  |  |  |
| Moderate or heavy calcification | 1.24 | 0.57–2.69 | 0.58 |  |  |  | 0.80 | 0.24–2.62 | 0.71 |  |  |  |
| Chronic total occlusion |  |  | 0.99 |  |  |  |  |  | 0.99 |  |  |  |
| Eccentric | 1.31 | 0.62–2.79 | 0.47 |  |  |  | 1.45 | 0.46–4.56 | 0.53 |  |  |  |
| Lesion bending | 2.93 | 1.38–6.21 | 0.0005 | 2.59 | 1.18–5.70 | 0.02 | 4.70 | 1.52–14.6 | 0.007 | 5.33 | 1.59–17.8 | 0.006 |
| True bifurcation | 1.17 | 0.53–2.56 | 0.70 |  |  |  | 0.83 | 0.22–3.05 | 0.78 |  |  |  |
| Ostial LCX stenting | 4.62 | 1.10–19.4 | 0.04 | 4.55 | 1.02–20.2 | 0.047 |  |  | 0.99 |  |  | 0.99 |
| Ostial RCA stenting | 7.99 | 1.09–58.7 | 0.04 | 5.72 | 0.70–46.8 | 0.10 |  |  | 0.99 |  |  | 0.99 |
| Bifurcation 2-stent approach | 1.22 | 0.17–9.00 | 0.85 |  |  |  |  |  | 0.99 |  |  |  |
| Minimum stent size of <3.0 mm | 0.74 | 0.35–1.56 | 0.43 |  |  |  | 0.79 | 0.25–2.50 | 0.69 |  |  |  |
| Total stent length of >28 mm | 1.55 | 0.72–3.34 | 0.26 | 1.39 | 0.61–3.15 | 0.43 | 0.24 | 0.03–1.89 | 0.18 | 0.22 | 0.03–1.79 | 0.22 |
| Minimum stent area of <5.0 mm^2^ | 0.90 | 0.42–1.89 | 0.77 | 0.69 | 0.40–1.84 | 0.69 | 0.27 | 0.06–1.25 | 0.09 | 0.27 | 0.06–1.26 | 0.09 |
| Follow up LDL-C <100 mg/dL | 0.63 | 0.30–1.32 | 0.22 | 0.71 | 0.28–1.80 | 0.47 | 0.23 | 0.07–0.72 | 0.01 | 0.26 | 0.08–0.86 | 0.03 |

TLR, target lesion revascularization; HR, hazard ratio; CI, confidence interval; LCX, left circumflex artery; RCA, right coronary artery.
